# Infectious etiology and indicators of malabsorption or intestinal injury in childhood diarrhea

**DOI:** 10.7705/biomedica.6913

**Published:** 2024-03-31

**Authors:** Adson Santos Martins, Samara Alves Santos, Claudia Alves da Silva Lisboa, Tânia Fraga Barros, Tereza Cristina Medrado Ribeiro, Hugo da Costa-Ribeiro, Ângela Peixoto de Mattos, Patrícia Silva de Almeida Mendes, Carlos Maurício Cardeal Mendes, Edna Lúcia Souza, Ana Lúcia Moreno Amor, Neci Matos Soares, Márcia Cristina Aquino Teixeira

**Affiliations:** 1 Programa de Pós-Graduação em Farmácia, Universidade Federal da Bahia, Salvador, Brasil Universidade Federal da Bahia Universidade Federal da Bahia Salvador Brazil; 2 Laboratório de Análises Clínicas do Hospital Geral de Salvador - Hospital do Exército, Salvador, Brasil Laboratório de Análises Clínicas do Hospital Geral de Salvador Hospital do Exército Salvador Brasil; 3 Faculdade de Farmácia, Universidade Federal da Bahia, Salvador, Brasil Universidade Federal da Bahia Faculdade de Farmácia Universidade Federal da Bahia Salvador Brazil; 4 Hospital Universitário Professor Edgard Santos - HUPES, Universidade Federal da Bahia, Salvador, Brasil Universidade Federal da Bahia Hospital Universitário Professor Edgard Santos - HUPES Universidade Federal da Bahia Salvador Brazil; 5 Instituto de Ciências da Saúde, Universidade Federal da Bahia, Salvador, Brasil Universidade Federal da Bahia Universidade Federal da Bahia Salvador Brazil; 6 Faculdade de Medicina da Bahia, Universidade Federal da Bahia, Salvador, Brasil Universidade Federal da Bahia Faculdade de Medicina da Bahia Universidade Federal da Bahia Salvador Brazil; 7 Centro de Ciências da Saúde, Universidade Federal do Recôncavo da Bahia, Santo Antônio de Jesus, Brasil Universidade Federal do Recôncavo da Bahia Centro de Ciências da Saúde Universidade Federal do Recôncavo da Bahia Santo Antônio de Jesus Brazil

**Keywords:** diarrheal disease, infectious, children, enterobacteria, enterovirus, Entamoeba histolytica, Giardia duodenalis, Blastocystis, steatocrit, diarrea infecciosa, niños, enterobacterias, enterovirus, Entamoeba histolytica, Giardia duodenalis, *Blastocystis* rotavirus, esteatocrito

## Abstract

**Introduction.:**

The multifactorial etiology of gastroenteritis emphasizes the need for different laboratory methods to identify or exclude infectious agents and evaluate the severity of diarrheal disease.

**Objective.:**

To diagnose the infectious etiology in diarrheic children and to evaluate some fecal markers associated with intestinal integrity.

**Materials and methods.:**

The study group comprised 45 children with diarrheal disease, tested for enteropathogens and malabsorption markers, and 76 children whose feces were used for fat evaluation by the traditional and acid steatocrit tests.

**Results.:**

We observed acute diarrhea in 80% of the children and persistent diarrhea in 20%. Of the diarrheic individuals analyzed, 40% were positive for enteropathogens, with rotavirus (13.3%) and *Giardia duodenalis* (11.1%) the most frequently diagnosed. Among the infected patients, occult blood was more evident in those carrying pathogenic bacteria (40%) and enteroviruses (40%), while steatorrhea was observed in infections by the protozoa *G. duodenalis* (35.7%). Children with diarrhea excreted significantly more lipids in feces than non-diarrheic children, as determined by the traditional (p<0.0003) and acid steatocrit (p<0.0001) methods. Moreover, the acid steatocrit method detected 16.7% more fecal fat than the traditional method.

**Conclusions.:**

Childhood diarrhea can lead to increasingly severe nutrient deficiencies. Steatorrhea is the hallmark of malabsorption, and a stool test, such as the acid steatocrit, can be routinely used as a laboratory tool for the semi-quantitative evaluation of fat malabsorption in diarrheic children.

## Resumen

**Introducción.:**

La etiología multifactorial de la gastroenteritis enfatiza la necesidad de usar diferentes métodos de laboratorio para identificar o excluir agentes infecciosos y evaluar la gravedad de la enfermedad diarreica.

**Objetivo.:**

Diagnosticar la etiología infecciosa de la diarrea en niños y evaluar algunos marcadores fecales asociados con la integridad intestinal.

**Materiales y métodos.:**

Se estudiaron 45 niños con enfermedad diarreica, en los cuales se evaluaron la presencia de enteropatógenos y los marcadores de malabsorción. Se analizaron las muestras fecales de 76 niños, mediante las pruebas de esteatocrito tradicional y esteatocrito ácido, para la cuantificación de la grasa.

**Resultados.:**

Se observó diarrea aguda en el 80 % de los niños y diarrea persistente en el 20 %. De los individuos con diarrea, el 40 % fue positivo para enteropatógenos; los más diagnosticados fueron rotavirus (13,3 %) y *Giardia duodenalis* (11,1 %). Entre los pacientes infectados, la sangre oculta fue más evidente en aquellos portadores de bacterias patógenas (40 %) o enterovirus (40%), mientras que la esteatorrea se observó en infecciones por el protozoo *G. duodenalis* (35,7 %). Los niños con diarrea excretaron significativamente más lípidos en las heces que aquellos sin diarrea, según lo determinado por los métodos de esteatocrito tradicional (p<0,0003) y esteatocrito ácido (p<0,0001).

**Conclusiones.:**

La diarrea infantil puede provocar deficiencias graves de nutrientes. La esteatorrea es distintiva de la malabsorción intestinal y puede detectarse mediante la estimación del esteatocrito ácido. Esta prueba podría utilizarse de forma rutinaria como una herramienta de laboratorio para la evaluación semicuantitativa de la malabsorción de grasas en niños con diarrea.

Diarrhea is the third most common cause of illness in children in developing countries and accounts for about one-third of all hospitalizations among children under five years of age ^1^^,^^2^. Diarrhea is characterized by an increase in the number of bowel movements with excretion of soft or liquid feces. According to the period of diarrhea resolution, it can be classified as acute (up to two weeks), persistent (from two to four weeks), and chronic (episodes lasting more than four weeks) ^2^^,^^3^. In poor countries, diarrhea mainly affects individuals with reduced sanitation and hygiene conditions. It is often caused by infectious agents such as viruses, bacteria, or parasites, primarily transmitted through the fecal-oral route, carried by water or contaminated food ^4^^-^^6^.

Recently, a global pediatric diarrhea surveillance network enrolled children under five years, hospitalized with diarrhea from 28 low-income and middle-income countries, testing stool specimens by quantitative PCR for 16 infectious agents. Of the 5,465 samples tested, rotavirus was the leading cause of diarrhea (33.3%), followed by *Shigella* spp. (9.7%), norovirus (6.5%) and adenovirus (5.5%) ^7^. When considering only Central and South America, the most frequent pathogens in diarrheal disease were *Shigella* spp. (19.2%) and norovirus (22.2%) ^7^. Conversely, a molecular study conducted in Brazil showed that *Escherichia coli* was the leading infectious agent, detected in 71/110 (65%) children with diarrhea ^8^.

Inflammation due to intestinal infections may alter the microvillous architecture and physiology, leading to nutrient malabsorption, including lipids. If untreated, fat malabsorption may result in malnutrition, growth failure, and deficiencies of fat-soluble vitamins A, E, D, and K ^9^. Fat loss in feces can be evaluated using qualitative methods, such as SUDAN III ^10^ (rendering low accuracy) and quantitative or semi-quantitative tests ^11^. The gold standard test for fecal fat determination is the van de Kamer method, which consists of collecting the feces over 72 hours and determining the fecal fat extracted with petroleum ether. Because of the need for a high amount of fecal material, specific laboratory infrastructure, and the time-consuming protocol, the van de Kamer method use is not feasible in routine laboratories. Another method to evaluate the fat content in feces is the traditional or acid steatocrit ^12^. Some studies have reported its clinical applicability in the semi-quantitative assessment of steatorrhea degree in preterm infants and in several pediatric conditions, like coeliac disease, cystic fibrosis, and acute and chronic diarrhea ^11^^-^^13^.

This study aimed to evaluate the infectious etiology of diarrhea in hospitalized children to determine some indicators of intestinal malabsorption, and to analyze the performance of steatocrit tests (traditional and acid) in the fecal fat estimation of children with diarrhea.

## Materials and methods

### Study design and population

We conducted this cross-sectional study at the pediatric center of *Professor Edgard Santos University Hospital* and the *Laboratório de Análises Clínicas* of the *Faculdade de Farmácia, Universidade Federal da Bahia,* Brazil.

Children aged 0-5 years were selected by convenience sampling between 2016 and 2017 and grouped as follows: 45 inpatients with diarrheal illness (for pathogens and functional coprology evaluation) and 76 children, matched by age, exclusively for the latter comparison between traditional and acid steatocrit. The latter study sample consisted of 48 children (outpatients) with diarrhea and 28 apparently healthy children as a control group for fecal fat loss assessment. Children's families completed a questionnaire about socioeconomic and sanitation conditions, diarrhea duration, and other gastrointestinal symptoms.

### Evaluation of fat, reducing substances and blood in fecal samples

The intestinal function of children with diarrhea included the steatocrit estimation for lipids detection, Benedict's reaction for reducing substances ^14^, and an immunochromatographic test for occult blood (Dialab® Gmbh, Wiener Neudorf, Austria). Traditional steatocrit was carried out through microcentrifugation of aqueous fecal homogenate, according to a previous report ^15^. The acid steatocrit method was similarly performed, except for perchloric acid addition (1/5 v/v) to the fecal homogenate ^16^. In this study, the steatocrit reference value was 3%, according to Cueto Rua, *et al.*^13^.

A control group for fat loss evaluation included 28 non-diarrheic children (described above in the *Study design and population* section), routinely seen at the *Laboratório de Análises Clínicas.* This group matched diarrheic patients' age and family income but lacked previous gastrointestinal diseases.

### Identification of intestinal pathogens

Stool samples were analyzed using the following laboratory methods, depending to the infectious agent to be detected: a) direct examination, zinc sulfate (solution density of 1.18 g/ml), centrifugal flotation ^17^, sedimentation by centrifugation in water ^18^, and modified Ziehl-Neelsen staining ^19^ for helminths and protozoa diagnosis; b) ELISA for coproantigen detection of *G, duodenalis, Cryptosporidium* sp., and *Entamoeba histolytica* (Wampole II *Cryptosporidium, Giardia* II, and *E. histolytica* II, TECHLAB, Blacksburg, VA, USA); c) routine stool culture for pathogenic enterobacteria isolation, and d) rapid immunochromatographic test for rotavirus and adenovirus detection (RIDA^®^ Quick Rotavirus / Adenovirus Combi, Germany), according to manufacturer's instructions.

### Statistical analysis

We performed the statistical analysis with the GraphPad InStat program (GraphPad Software, Inc., San Diego, California, USA). We used the chi square test to analyze frequencies of infections by enteric pathogens, intestinal malabsorption markers or injury, and steatorrhea in children discriminated by the method. For steatocrit performance, we compared the values of eliminated fat by children from diarrhea and asymptomatic groups using the Student t test. A probability value under 0.05 was considered statistically significant.

### Ethical considerations

The Research Ethics Committee of the *Universidade Federal da Bahia, Escola de Enfermagem,* approved this study, protocol # 907.867. We sent all laboratory results to the children's parents. Participants who tested positive for pathogenic intestinal microorganisms were treated appropriately by their respective pediatricians.

## Results

### Evaluation of intestinal infections, other gastrointestinal symptoms, and malabsorption markers or damage in children with diarrhea

All examined children used public health services and came from low-income families, with 71.1% receiving up to one minimum Brazilian wage (around USD$ 260,00 per month), and only 26.4% of their parents had completed or were attending high school (table 1). Despite the low family income, most children (>95.0%) had basic sanitation conditions and access to piped water in their residences, which is compatible with inhabitants of urban and peri-urban areas (94.2% of residential areas).

**Table 1:** Demographic, socioeconomic and environmental sanitation characteristics of the studied population

Variables	Children [n (%)]		
	Diarrhea*	Asymptomatic	Total
	93 (76.9)	28 (23.1)	121 (100.0)
Gender			
Female	41 (44.1)	13 (46.4)	54 (44.6)
Male	52 (55.9)	15 (53.6)	67 (55.4)
Age (years) 0 ≤ 2	55 (59.1)	14 (50.0)	69 (57.0)
> 2 ≤ 5	38 (40.9)	14 (50.0)	52 (43.0)
Area of residence			
Urban/peri-urban	87 (93.6)	27 (96.4)	114 (94.2)
Rural	6 (6.4)	1 (3.6)	7 (5.8)
Highest education level of the mother			
None	3 (3.2)	0 (0.0)	3 (2.5)
Elementary school	53 (57.0)	12 (42.9)	65 (53.7)
High school	22 (23.7)	10 (35.7)	32 (26.4)
College	15 (16.1)	6 (21.4)	21 (17.3)
Monthly income (minimum wage)			
≤ 1	67 (72.0)	19 (67.9)	86 (71.1)
> 1 ≤ 2	20 (21.5)	7 (25.0)	27 (22.3)
> 2	6 (6.5)	2 (7.1)	8 (6.6)
Access to water and sanitation			
Piped water	89 (95.7)	28 (100.0)	117 (96.7)
Sewage system	89 (95.7)	28 (100.0)	117 (96.7)
Bathroom with sink	88 (94.6)	27 (96.4)	115 (95.0)

Among the 45 children hospitalized with diarrheal disease, 36 (80%) presented acute. and 9 (20%) persistent diarrhea at the time of the fecal sample collection (table 2). Of the 45 children, 18 (40.0%) were infected by enteric pathogens. Intestinal viruses predominated (15.6%), followed by protozoa (13.3%) and enterobacteria (11.1%) (table 2). Single infections by rotavirus were more frequently detected (5/7; 71.4%; p<0.05) than adenovirus (1/7; 14.3%), with one child co-infected with the two enteroviruses. The stool culture revealed five children (11.1%) positive for pathogenic enterobacteria (table 2). Most children with diarrhea diagnosed with parasitic infections had *G. duodenalis* (5/6; 83%; (p<0.05). Four children had single infections, and one was coinfected with *E. histolytica.* One child presented *Blastocystis* spp. in feces (table 2). We observed other symptoms besides diarrhea in infected and noninfected individuals. Among the 18 children positive for infectious agents, the cases with fever were more related to the presence of enterovirus, vomiting events to bacteria, and coughing episodes to parasitic infections (table 2).

**Table 2:** Enteropathogens evaluation, symptoms and markers of intestinal function and integrity in 45 hospitalized children with diarrheic illness in a pediatric hospital in Salvador, Bahia

	Children	Total	Type of diarrhea			Other symptoms			Intestinal markers of malabsorption or injury		
			n (%)			n (%)			n (%)		
	Total	45 (100)	Acute	Persistent	Fever	Vomit	Inappetence	Cough	Hemoglobin	Lipids*	Carbohydrates
			36 (80.0)	9 (20.0)	29 (64.4)	18 (40.0)	19 (42.2)	11 (24.4)	12 (26.7)	28b (62.2)	5 (11.1)
	Non-infected	27 (60.0)	21 (77.8)	6 (22.2)	21 (77.8)	11 (40.7)	17 (62.9)	6 (22.2)	7 (25.9)	14 (51.8)	3 (11.1)
	Infected	18 (40.0)	15 (83.3)	3 (16.7)	8 (44.4)	7 (38.9)	2 (11.1)	5 (27.8)	5 (27.8)	14 (77.8)	2 (11.1)
	Protozoa	6 (13.3)	5 (83.3)	1 (16.7)	2 (33.3)	2 (33.3)	0 (0.0)	3 (50.0)	1 (16.7)	6 (100.0)	0 (0.0)
	*Giardia duodenalis*	4^a^ (8.9)	4 (100.0)	0 (0.0)	2 (50.0)	2 (50.0)	0 (0.0)	1 (100.0)	0 (0.0)	4 (100.0)	0 (0.0)
	*Giardia duodenalis* +	1 (2.2)	1 (100.0)	0 (0.0)	0 (0.0)	0 (0.0)	0 (0.0)	1 (100.0)	0 (0.0)	1 (100.0)	0 (0.0)
	*Entamoeba histolytica* *Blastocystis* sp.	1 (2.2)	0 (0.0)	1 (100.0)	0 (0.0)	0 (0.0)	0 (0.0)	1 (100.0)	1 (100.0)	1 (100.0)	0 (0.0)
	Enterovirus	7 (15.6)	6 (85.7)	1 (14.3)	4 (57.1)	2 (28.6)	1 (14.3)	1 (14.3)	2 (28.6)	5 (71.4)	2 (28.6)
	Rotavirus	5^a^ (11.1)	5 (100.0)	0 (0.0)	3 (60.0)	1 (20.0)	0 (0.0)	1 (20.0)	2 (40.0)	4 (80.0)	1 (20.0)
	Adenovirus	1 (2.2)	0 (0.0)	1 (100.0)	1 (100.0)	0 (0.0)	0 (0.0)	0 (0.0)	0 (0.0)	1 (100.0)	0 (0.0)
	Rotavirus + Adenovirus	1 (2.2)	1 (100.0)	0 (0.0)	0 (0.0)	1 (100.0)	1 (100.0)	0 (0.0)	0 (0.0)	0 (0.0)	1 (100.0)
	Enterobacteria	5 (11.1)	4 (80.0)	1 (20.0)	2 (40.0)	3 (60.0)	1 (20.0)	1 (20.0)	2 (40.0)	3 (60.0)	0 (0.0)
	*Escherichia coli*	4 (8.9)	4 (100.0)	0 (0.0)	1 (25.0)	2 (50.0)	1 (25.0)	1 (25.0)	2 (50.0)	3 (75.0)	0 (0.0)
	*Salmonella* spp.	1 (2.2)	0 (0.0)	1 (100.0)	1 (100.0)	1 (100.0)	0 (0.0)	0 (0.0)	0 (0.0)	0 (0.0)	0 (0.0)

* Determined by steatocrit; cut-off limit = 3.0% of fecal fat (Cueto Rua, *et al.,* 2006).

Statistically significant differences between groups: ^a^p<0.05 and ^b^p<0.001, x^2^ test

Among the markers of intestinal injury, lipids loss in feces occurred more frequently in children with diarrhea (28/45; 62.2%) compared to hemoglobin loss (12/45; 26.7%) or carbohydrates (5/45; 11.1%; p<0.001) (table 2). Steatorrhea was proportionally more frequent in infected individuals (14/18; 77.8%) than in non-infected individuals (14/27; 51.8%), although without statistical significance (p>0.05). There were no differences in the frequency of fecal occult blood of children infected or not by intestinal pathogens. Five children tested positive for carbohydrates in feces. Of these, two were infected by enterovirus (table 2).

### Comparison between ratios of fecal fat loss measured by traditional and acid steatocrit in children with diarrhea

Some authors have reported greater efficacy of fecal fat extraction in acidic media ^16^ for the semi-quantitative determination of fat loss in feces (acid steatocrit). Thus, we compared the traditional steatocrit with the acid steatocrit in 48 samples from children with diarrhea using other samples than those used to assess infectious etiology since previous material was insufficient to perform various tests or, in some cases, inadequately preserved for routine stool culture.

**Figure 1:**
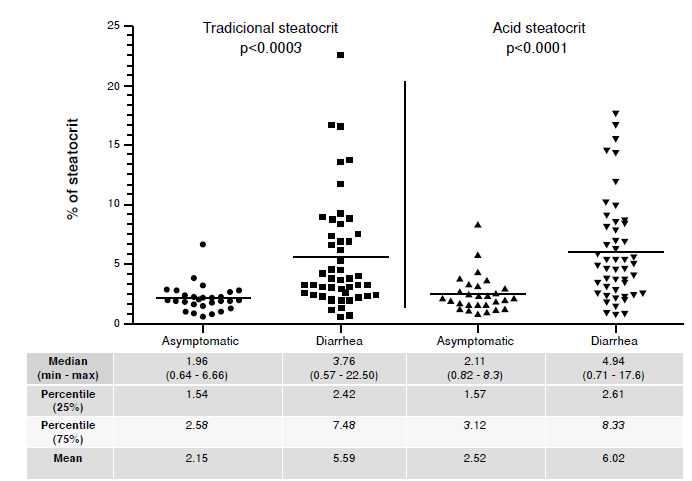
Comparison between traditional and acid steatocrit rates of fecal samples from children with diarrhea (n=48) and asymptomatic (n=28)

As controls, we used 28 fecal samples from healthy children to evaluate the fecal fat rate. The children with diarrhea had a mean of 5.59% for traditional steatocrit and 6.02% for acid steatocrit (median of 3.76% and 4.94%, respectively). Fecal samples from the asymptomatic children, used as controls, presented a mean of 2.15% for traditional steatocrit and 2.52% for the acid one (figure 1).

The observed steatocrit rates were statistically different between the diarrheic and asymptomatic groups, both by traditional (p<0.0003) and acidic method (p<0.0001) (figure 1). Percentages of fecal fat detected by the acid steatocrit method were 16.7% higher than those obtained by the traditional. Nonetheless, within the same group of children (diarrheic or asymptomatic), we did not observe statistical significance between the fat values obtained by traditional or acid steatocrit.

Similar to steatocrit rates, the frequency of children with steatorrhea in the diarrheal disease group compared to asymptomatic children was also significant, regardless of the steatocrit method used, if traditional (p<0.00001) or acid (p<0.0001) (table 3). Considering a normal reference value up to 3%, 32/48 (66.7%) diarrheic children were positive by the traditional and 35/48 (72.9%) by the acid steatocrit method, while 3/28 (10.7%) and 7/28 (25%) asymptomatic children were positive by the traditional and acid methods, respectively (table 3). Although we noticed a slight increase in the frequency of children with steatorrhea using the acid method, this was neither statistically significant for the diarrhea (p=0.656) nor the asymptomatic group (p=0.295) (table 3).

**Table 3:** Positivity for fecal fat loss by traditional and acid steatocrit in children with and without diarrhea

Steatorrhea	Steatocrit results					
	n (%)					
	Traditional		*p	Acid**		*p
	Asymptomatic	Diarrhea	0.00001	Asymptomatic	Diarrhea	0.0001
Negative	25 (89.3)	16 (33.3)		21 (75.0)	13 (27.1)	
Positive	3 (10.7)	32 (66.7)		7 (25.0)	35 (72.9)	
Total	28 (100.0)	48 (100.0)		28 (100.0)	48 (100.0)	

Positivity for fecal fat loss by steatocrit (reference value ≥ 3%)

* p-value comparing steatorrhea positivity between children with and without diarrhea

** No significant statistical difference was observed in the frequency of steatorrhea in children when detected by the acid steatocrit compared to the traditional method (p=0.295 for the asymptomatic group and p=0.656 for the diarrhea group).

## Discussion

In the management of children with diarrhea, it is essential to establish the enteric segment affected, the mechanisms involved (physiopathology), the chronology of the diarrheal process (acute, persistent, or chronic diarrhea), and the potential enteritis infectious etiology ^20^. According to Andrade and Fagundes-Neto ^21^, the rates of progression from an acute episode to persistent diarrhea range from 3% to 28% in children under five years of age. This rate depends on numerous reasons, such as enteropathogenic agents isolated in feces, seasonality, geographic aspects, socioeconomic and educational conditions, and levels of environmental sanitation ^21^. In this study, we found nine cases of persistent diarrhea. Persistent or chronic diarrheal disease has a high impact on morbidity and mortality rates in pediatric populations in developing countries. More than 50% of diarrheal deaths in these places are associated with persistent diarrheal syndrome ^2^^,^^5^.

In developing countries, low socioeconomic conditions are usually linked to gastrointestinal infections caused by parasites, viruses, and bacteria, especially during childhood ^22^^,^^23^. In this study, the analysis of the socioeconomic aspects of the children with diarrhea showed that they were from families with low educational and income levels, both variables usually associated with intestinal pathogen infections in children ^23^^-^^25^.

Rotavirus is considered the most important etiological agent of severe diarrhea in childhood worldwide ^26^. In this work, 7/45 (15.6%) diarrheic children were diagnosed with enteroviruses, predominating rotavirus infections. Of the five children testing positive for fecal blood, two had rotavirus. Other studies conducted with pediatric patients with diarrhea in the northeastern region of Brazil showed high frequencies (up to 19%) of rotavirus, with the presence of fever, vomiting, and dehydration ^27^^,^^28^. This virus has a higher occurrence in children between four and nine months of age, making it one of the most frequent causes of acute abdominal pain in this age group. One study measuring the occurrence of adenovirus in fecal samples from children with acute gastroenteritis in Belém demonstrated 3.7% (13/380) positivity for adenoviruses ^29^, a similar frequency found in our study. It is important to note that we did not perform norovirus diagnosis nor use molecular biology tools to detect rotavirus, which could increase the positivity rate of enterovirus infections in the population studied. Norovirus is considered the second cause of gastroenteritis after rotavirus infection, and epidemiological studies show that norovirus infection is frequently associated with outpatient consultations due to gastrointestinal symptoms ^30^.

Infections by helminths and intestinal protozoa cause a wide range of symptoms associated with the gastrointestinal tract, depending on host demographic, socioeconomic, and immunological factors of the hosts ^31^^,^^32^. In this work, six children with diarrhea had infections by intestinal protozoa. Four of these children had single infections by *G. duodenalis,* two reported episodes of vomiting and fever (symptoms that can be associated with giardiasis), and one child had a concomitant infection by *G. duodenalis* and *E. histolytica.* Belloto *et al.* ^*(*^^33^ found *G. duodenalis* in 47 (15.16%) and *E. histolytica* in two (0.64%) of the 310 schoolchildren studied in São Paulo. Some reports have associated *G. duodenalis* infections with childhood diarrhea ^21^^,^^34^^,^^35^. However, studies developed by our group found *G. duodenalis* mostly in asymptomatic children, which seems more common in endemic countries such as Brazil ^36^^,^^37^, and only one child with persistent diarrhea had a single infection by *Blastocysts* sp. Most *Blastocysts* sp. infected patients around the world are asymptomatic, but when symptoms persist without other causes, anti-parasite treatment is recommended ^38^.

Epidemiological studies on species-specific *Entamoeba* infections are limited due to the morphological resemblance of *Entamoeba histolytica* with non-pathogenic *E. dispar* and *E. moshkovskii.* Specific methods based on molecular techniques like PCR and fecal detection of *E. histolytica* antigens are required to make a reliable diagnosis of *E. histolytica* intestinal infections. A study in Pernambuco analyzed 213 stool samples, with 10 (4.7%) positive for *E. histolytica/E. dispar/E. moshkovskii* complex, but when tested by ELISA for *E. histolytica* antigens, all samples were negative ^39^.

Previous studies by our group in Salvador found 788/52,704 (3.4%) and 273/55,218 (0.49%) positive fecal samples for *E. histolytica/E. dispar/E. moshkovskii* complex by microscopic examination. The group randomly analyzed some amoeba-positive stools by PCR and ELISA. All of them were negative for *E. histolytica* and positive for *E. dispar*^40^. Moreover, the evaluation of specific antibodies in sera by ELISA detected a seropositivity of 8.9% (8/90). These results point out the absence or very low prevalence of *E, histolytica* in asymptomatic carriers in the evaluated population of Salvador, and the antibody production induced by *E. dispar* infections ^40^^,^^41^. Nonetheless, our study finding of *E. histolytica* in one child with diarrhea demonstrates the need for specific laboratory diagnosis of this parasite, even in areas with low transmission.

A study in Brazil with children groups reported an approximate frequency of 10% of diarrheic bacterial infections ^42^. This result is similar to that found herein (11.1%). Moura *et al.*^43^ evaluated 140 stools of diarrheic children in Pernambuco with socioeconomic conditions comparable to those of the population in our study in Salvador. They found 9 (6.4%) samples positive for enteropathogenic and invasive *Escherichia coli* and 3 (2.1%) for *Salmonella* spp. In this study, two of the four patients who tested positive for *E. coli* had blood in their stools. Furthermore, the child with *Salmonella* spp. had persistent diarrhea, fever, and episodes of vomiting, common symptoms of salmonellosis. Anal fissures are often related to fecal blood, followed by infectious diseases ^44^. However, none of the participating children presented anal fissures, as observed in medical records.

Among the 45 children with diarrheal disease, five excreted reducing substances in their feces: one had rotavirus infection, one adenovirus infection, and the remaining three were negative for enteric pathogens. Viral replication in the intestinal villi epithelium of the jejunum can induce a process of malabsorption, mainly due to a transient decrease of disaccharidases ^14^^,^^45^. Reducing substances present in stool indicates carbohydrate intolerance, usually secondary to a viral illness. On the other hand, the sugar loss in the feces of non-infected patients may be related to the timedecrease of the fecal bolus in the intestine due to the diarrheal process, generating a reduced absorption of several nutrients, including carbohydrates.

Fecal fat examination generally aims to establish a more objective diagnostic pattern of malabsorption origin (infectious, celiac, or other autoimmune diseases or cystic fibrosis) and in the therapeutic approach. Fecal fat loss estimation usually requires quantitative or semi-quantitative tests. The gold standard test for fecal fat quantitative determination is the van de Kamer test. However, despite its low cost, collecting all stools over 72 hours is inconvenient and difficult to perform with constipated patients, neonates, and infants. In addition, this method requires the infrastructure and reagents for fat extraction and quantification by chemical processes that are not always available in routine laboratories ^15^^,^^46^^,^^47^.

Despite the use of the traditional steatocrit method in the evaluation of fecal fat, this test also presents some limitations, such as: the reading of the fat layer when the patient is eliminating insignificant or even undetectable fat amounts, or the observer's inexperience in delimiting the layers for a proper reading of the fat rates. However, performing the technique in triplicate, correctly homogenizing the sample, centrifuging the material in a defined time ^15^, and even using an acidic media for a more effective fecal fat extraction, as reported ^16^, can reduce execution error.

In this study, we observed a significant difference in the rates of fat loss in children with diarrhea compared to the asymptomatic children. This effect was evident when using acid steatocrit (p<0.0001). Other authors reported fat malabsorption occurrence in children with diarrhea, as observed in this study ^20^^,^^21^^,^^45^. Due to the higher sensitivity of the acid steatocrit in contrast to the classic steatocrit, this method can be useful to rule out steatorrhea as a screening laboratory tool, avoiding subjecting patients to more laborious and timeconsuming techniques such as the van de Kamer test ^16^^,^^47^. It is worth mentioning the increased number of steatorrhea positive cases in asymptomatic children found in our study when using the acid method. A follow-up with consecutive determinations or a quantitative evaluation of fecal fat would be advisable to discard false positive cases of steatorrhea.

Infectious agents and dietary errors are the principal causes of diarrhea in children, and infections by enteropathogens are the most relevant etiology in developing countries ^27^^,^^48^. In this context, methods used to identify infectious agents and to study intestinal malabsorption markers in health services can help to diagnose intestinal diseases, to promote prompt treatment, and to improve prognosis in childhood. The multifactorial etiology of gastroenteritis emphasizes the need for different laboratory methods to identify or exclude infectious agents, to determine markers of intestinal malabsorption, and to evaluate the severity of diarrheal disease.

A determinant limitation of this study was the sample size to evaluate the different pathogens and intestinal integrity markers in children with diarrhea. Among the difficulties encountered, the most important was the reduced amount of feces collected from hospitalized children and their inadequate preservation until laboratory analysis, hampering the use of many samples during the study. Despite the small sample size, the obtained results were similar to those of other studies conducted in Brazil ^27^^-^^29^^,^^34^^,^^35^^,^^40^^-^^42^. Moreover, in another study carried out by our group, from January 2011 to June 2012, we analyzed parasitic infections in 151 children with diarrhea from Salvador and we found a relatively low frequency of *Cryptosporidium* (4.6%) and *E. histolytica* (3.3%), and the absence of *Cyclospora cayetanensis* and *Cystoisospora belli*^18^. Therefore, a larger sample size would be necessary to find any of these latter parasites in such a specific population.

In conclusion, this study highlights the importance of monitoring childhood diarrhea by different laboratory methods, including acid steatocrit measurement as an alternative tool for the semiquantitative evaluation of fecal fat.
